# Antimicrobial light-based interventions for management of oral infections: a mini review

**DOI:** 10.3389/froh.2026.1981642

**Published:** 2026-10-14

**Authors:** Gal Hiltch, Liviu Steier, Katia Rupel, Giulia Ottaviani, José Antonio Poli De Figueiredo

**Affiliations:** 1 Department of Dentistry, Federal University of Rio Grande do Sul—UFRGS, Porto Alegre, Brazil, Porto Alegre, Brazil; 2 Department of Preventive and Restorative Sciences, Dental School, University of Pennsylvania—UPENN, Philadelphia, PA, United States; 3 Department of Medicine, Surgery and Health Sciences, University of Trieste, Trieste, Italy; 4 Department of Morphological Sciences, Federal University of Rio Grande do Sul—UFRGS, Porto Alegre, Brazil

**Keywords:** antimicrobial blue light, antimicrobial resistance, antimicrobial therapy, oral infection, photothermal therapy (PPT)

## Abstract

Limitations of conventional disinfectants and rising antimicrobial resistance have accelerated the development of light-based, resistance-free interventions. This mini-review describes the mechanisms and evidence for antimicrobial photodynamic therapy (aPDT), antimicrobial blue light (aBL), antimicrobial selective photothermolysis (aSP) and photothermal therapy (PTT). Unlike antibiotics, light-based interventions avoid inducing microbial resistance or disturbing the host microflora. Interventions were evaluated across endodontics, periodontal and peri-implant disease, oral mucosal infections, and dental caries. Current literature is heavily skewed towards aPDT, leaving other interventions underexplored despite their mechanistic promise. aPDT is currently the only one with systematic reviews and meta-analyses, whereas evidence for aBL and PTT remains confined to *in-vitro* and preclinical studies. In periodontitis, aPDT achieved statistical significance, but clinical improvements were negligible and below meaningful thresholds, leading official guidelines from major bodies against its routine use. Clinical evidence currently supports aPDT only in oral candidiasis, where outcomes matched conventional treatments. aSP, tested only as pulsed Nd:YAG or CW diode adjuncts to debridement, was not superior to conventional methods, and selectivity has never been measured clinically. Promising directions are aBL, due to its selectivity for periodontopathogens and link to autofluorescence-based diagnosis, and PTT for its combined antimicrobial and regeneration capacity. Physical limiting factors for aPDT and aBL are shallow effective depth and oxygen dependence in anaerobic niches, and a narrow thermal safety margin for aSP and PTT. A recurring gap is lack of translation between microbial reduction and improved clinical outcomes across interventions, highlighting the need for multi-center trials based on clinically proven standardized protocols.

## 1 Introduction

Antimicrobial resistance (AMR) is a public health risk: roughly 4.7 million associated deaths in 2021 place it among the leading causes of death globally (1). Dental prescribing accounts for roughly 10% of global antibiotic consumption, contributing significantly to AMR strains (2). The elimination of biofilms and bacterial deposits infecting tissues plays a pivotal role in achieving a successful treatment. In endodontics, more than 35% of the root canal surfaces remain uninstrumented regardless of the applied technique (3). In periodontics, scaling and root planing (SRP) leaves residual calculus on up to 57% of surfaces (4). When treating oral candidiasis infections, fluconazole resistance now exceeds 50% among oral *Candida* isolates from ICU patients (5). Light-based interventions are a promising, “pills-free” option that avoids inducing microbial resistance and limits side effects. This review covers four light-based interventions: antimicrobial photodynamic therapy (aPDT) (6), antimicrobial blue light (aBL) (7), antimicrobial selective photothermolysis [aSP; a term introduced by Harris and Sulewski (8), not yet in general use] and photothermal therapy (PTT) (9).

The objectives of this review are to: (A) describe the mechanisms of each light-based modality; (B) critically appraise the highest available evidence across endodontic infections, periodontal and peri-implant disease, oral mucosal infections, and dental caries; and (C) identify key limitations and research priorities. Relevant literature was identified through PubMed/MEDLINE and the Cochrane Library together with current clinical guidelines, prioritizing the highest available level of evidence; this was a non-systematic, author-led selection. For each modality and domain, the level of evidence (*in-vitro*/ex-vivo, preclinical, clinical trial, systematic review) is stated in the text and graded in Table 1.

**Table 1:** Evidence summary by modality and clinical domain.

Clinical Domain	aPDT	aBL	aSP	PTT
Endodontics	**Level 1.** SR/MA: 91–100% bacterial reduction; no clinical outcome correlation; AAE/ESE: not recommended	**Level 5.** *Ex vivo* only; no SR; no standardized protocol	**Level 1.** Umbrella SR: low to critically low evidence; inferior or equivalent to irrigation alone; AAE/ESE: not recommended (pulsed Nd:YAG and CW diode pooled; selectivity not measured)	**Level 5.** *In vitro*/*ex vivo* only; no SR or clinical trials
Periodontitis/Peri-implant	**Level 1.** SR/MA: PD −0.37 mm, CAL +0.33 mm (sub-threshold); EFP: not recommended; 2024 Cochrane: very low certainty; peri-implantitis MA 2025: PPD −0.8 to −0.9 mm, low to moderate certainty; EFP 2023: not recommended for peri-implantitis	**Level 4.** *In vitro* + 1 clinical trial; no SR	**Level 1.** Overview SR: marginal benefit over SRP; AAP: marginal adjunctive benefit; EFP 2023: lasers not recommended for peri-implantitis (pulsed Nd:YAG and CW diode pooled; selectivity not measured); Nd:YAG-only MA: PD −0.46 mm, no CAL gain	**Level 5.** *In vitro*/animal only; no SR; no peri-implant study
Oral Mucosal Infections	**Level 1.** SR/MA: equivalent to antifungals; no resistance induction; natural PS (riboflavin, curcumin, hypericin) also effective. Herpes labialis: Level 2. Two small RCTs; no difference vs. acyclovir; SR found case reports only. Bacterial mucosal infections: not investigated	**Level 5.** *In vitro* only; no SR or RCT; ∼42-fold selectivity over keratinocytes demonstrated; enveloped viruses inactivated *in vitro*; no intraoral study	Not investigated (Candida and viruses lack NIR chromophore)	Not investigated (non-oral wound models only)
Dental Caries	**Level 1.** SR: bacterial reduction in 5/6 studies; no improvement in bond strength vs. conventional methods	**Level 5.** *In vitro* only; no SR; non-significant results on carious dentin	Not investigated	**Level 5.** Scoping review: 9 publications (3 animal, 6 lab); no clinical trials; >99% antibacterial efficacy *in vitro*

Evidence levels follow the Oxford Centre for Evidence-Based Medicine (OCEBM) 2011 treatment-benefits ladder (Level 1, systematic review of randomized trials; Level 2, individual randomized trial; Level 3, non-randomized controlled cohort study; Level 4, case-series or case-control studies; Level 5, mechanism-based or *in-vitro* reasoning); levels denote study design, not certainty of evidence, and cells marked not investigated have no level assigned. SR, systematic review; MA, meta-analysis; PD, probing depth; CAL, clinical attachment level; PS, photosensitizer; AAE, American Association of Endodontists; ESE, European Society of Endodontology; EFP, European Federation of Periodontology; AAP, American Academy of Periodontology.

## 2 Mechanisms and biological effects

aPDT depends on the photochemical triad of an exogenous non-toxic photosensitizer (PS), light of a specific wavelength, and molecular oxygen. After topical application and incubation of 5–10 min (10, 11), the PS is activated by light and transitions from its ground state to short-lived singlet excited state to triplet excited state. From the triplet state the PS undergoes two pathways resulting in the generation of reactive oxygen species (ROS; O₂⁻·, ·OH, H₂O₂). Through type I pathway, the PS reacts with a biomolecule initiating a cascade that transforms cellular components into a wave of destructive ROS (12). Through type II pathway, the excited PS triplet state reacts with ground-state oxygen molecule (^3^O₂) which is dissolved in the tissue, converting ordinary oxygen into singlet oxygen (^1^O₂). ROS oxidize multiple cellular components, with bacterial membrane oxidation having the most destructive consequences (6, 13).

The most clinically used PSs include methylene blue (MB) and toluidine blue O (TBO), activated by red light of 635–660 nm, and indocyanine green, activated by near-infrared light of 810 nm (13).

aBL (405–470) utilizes endogenous chromophores within the bacteria to generate ROS through Type I/II reaction mechanisms. aBL shows broad efficacy against bacteria, fungi, parasites, and viruses (7, 14–16). The principal chromophore is Protoporphyrin IX (PpIX), the penultimate heme precursor, with peak absorption at 405–410 nm (Soret band) and weaker bands at 500–635 nm (17). Irradiation at 405 nm excites PpIX to the triplet state, driving predominantly Type II ROS generation (7, 14). Periodontopathic anaerobic bacteria such as *Porphyromonas gingivalis*, highly depend on exogenous heme (Iron-PpIX) acquisition for their metabolism, as they lack the ability for its de-novo synthesis (8, 17). Following its acquisition from the host cells, the iron is extracted from heme, leaving free PpIX as a byproduct or stored on the outer membrane as black-pigmented. This metabolic pathway makes anaerobic periodontopathic bacteria intrinsically photosensitive to 405 nm and accounts for the characteristic red autofluorescence of these species under violet-blue light excitation (17, 18).

aSP differs from conventional laser debridement/vaporization. In contrast to Er:YAG and CO_2_ lasers operating at the far infrared (1,300–11,000 nm) and vaporizing water and tissue in its path, diode and Nd:YAG lasers operate at near infrared (700–1,100 nm) where human tissue is transparent but bacterial pigments (mainly porphyrins) absorb and destroy the pathogens even if they invaded a human cell (8). Selective photothermolysis requires a chromophore-specific wavelength and a pulse shorter than the target's thermal relaxation time. Currently, Nd:YAG lasers satisfy both conditions (∼100 *μ*s pulses, ∼1,000 W peak) (8) as diode lasers are typically continuous-wave and heat tissue non-selectively (Table 2).

**Table 2:** Mechanistic comparison of light-based interventions.

Parameter	aPDT	aBL	aSP	PTT
Chromophore source	Exogenous (PS applied)	Endogenous (PpIX, flavins)	Endogenous (PpIX)	Exogenous (nanoparticle PTA)
Primary wavelength(s)	635–660 nm (MB/TBO); 810 nm (ICG)	405–470 nm	810, 980, 1,064 nm	700–1,100 nm (NIR-CW)
Killing mechanism	ROS (Type I & II)	ROS (Type I & II)	Localized hyperthermia (pulsed)	Sustained hyperthermia (CW)
Oxygen dependence	Yes (reduced efficacy in hypoxia)	Yes (primarily Type II)	No	No
Depth of kill (basis)	∼23 μm in Pg biofilm *in vitro* (purpurin PS); light penetration 0.5–0.75 mm	90–150 μm (1/e light penetration, skin); measured kill 30–45 μm	2–3 mm (thermal simulation, periodontal ligament); no *in vivo* measurement	Variable (nanoparticle-dependent)
Selectivity	Low-moderate (PS uptake dependent)	High (PpIX-rich anaerobes)	High (absorption ratio 333 × at 810 nm, 43 × at 1,064 nm; hemin-dependent)	Moderate (PTA delivery-dependent)
Biofilm penetration	Limited	Limited	Not characterized	High (nanoparticles penetrate EPS)
Regenerative potential	Limited	Limited	Limited	Yes (<45 °C modulates host response)
Evidence maturity	Highest (clinical SR/MA)	Low (predominantly *in vitro*)	Moderate (Nd:YAG/diode adjunct SR/MA; selectivity not measured)	Lowest (preclinical)

PS, photosensitizer; PTA, photothermal agent; PpIX, protoporphyrin IX; CW, continuous wave; EPS, extracellular polymeric substance; SR, systematic review; MA, meta-analysis. Depth values from ref 8; basis stated per cell.

PTT employs exogenous photothermal agents (PTAs) that convert continuous-wave near-infrared (NIR) irradiation into localized heat. The temperature elevation disrupts bacterial cell membranes, increases its permeability, and leads to leakage of essential intracellular components (9). Nanoparticle PTAs penetrate the extracellular polymeric substance (EPS) matrix, where hyperthermia inactivates nucleic acids and proteins. Temperatures above 50 °C denature microbial components, while below 45 °C they modulate host immune responses and promote tissue regeneration (9).

## 3 Endodontic disinfection

The root canal system's complex anatomy protects biofilms from irrigants and instrumentation. *Enterococcus faecalis*, detected in 24%–77% of persistent infections, penetrates dentinal tubules and resists medicaments and starvation (19).

### 3.1 Evidence level: systematic reviews

aPDT for endodontic disinfection involves the introduction of TBO or MB into the root canal followed by diode laser, Er:YAG laser or Nd:YAG laser activation (20). Chrepa et al. reported 91.3%–100% bacterial load reduction with adjunct aPDT in the first systematic review (21). Three further systematic reviews confirmed the findings (22–24). Despite accumulating microbiological evidence, a translation gap exists between microbial reduction and clinical outcomes. Araújo et al. (25) reported the discrepancy in a systematic report, where only one of four clinical trials showed correlation between the bacterial reduction and improved clinical outcomes. A mechanistic explanation lies in the ∼23 μm depth of kill of aPDT in biofilm *in-vitro* (8) relative to *E. faecalis* tubule invasion to depths of several hundred micrometers (19), together with PS retention by the smear layer and oxygen depletion within the sealed canal. Reflecting this gap, neither the American Association of Endodontists (AAE) nor the European Society of Endodontology (ESE) S3-level guidelines currently recommend aPDT for endodontic disinfection (26, 27).

### 3.2 Evidence level: ex-vivo studies

Three ex-vivo studies report divergent results that track irradiation geometry: direct intracanal 410 nm micro-LED placement achieved bactericidal depths exceeding calcium hydroxide (28), direct lateral 445 nm dentin irradiation reduced *E. faecalis* by 77%–84% (29), whereas indirect 445 nm transillumination (540 J/cm^2^) produced no significant reduction at any dentin thickness (30). No standardized protocol, clinical trial or systematic review exists.

### 3.3 Evidence level: overview of systematic reviews

aSP using Nd:YAG (1,064 nm) and diode lasers (810–980 nm) as adjuncts to NaOCl irrigation has shown inconclusive results. A 2026 umbrella review of nine systematic reviews rated the evidence low to critically low (AMSTAR 2), with lasers consistently inferior or equivalent to irrigation alone (31). They further concluded that Nd:YAG carries a documented risk of dentinal microcrack formation. Neither the AAE nor the ESE currently supports routine clinical use (26, 27). The reviews pool pulsed Nd:YAG with CW diode arms and report no pulse parameters or selectivity endpoints, so they test laser-assisted irrigation rather than selective photothermolysis as a mechanism.

### 3.4 Evidence level: in-vitro and ex-vivo studies

PTT in endodontic disinfection remains at an early preclinical stage, with no clinical trials or systematic reviews. Available evidence is limited to a small number of *in-vitro* and ex-vivo studies exploring nanomaterials with irrigants and intracanal dressings, to eliminate *E. faecalis* biofilms (9). A 2,025 review reported high anti-biofilm efficacy without loss of dentin strength (9).

## 4 Periodontal and peri-implant disease

Severe periodontitis is a leading cause for multiple tooth-loss, and peri-implant mucositis and peri-implantitis is estimated to affect 43%–46% and 19%–22% of implant patients, respectively (32). Since the primary etiological factor for both are bacterial biofilms, surface debridement and decontamination is pivotal to successfully treat the diseases (33).

### 4.1 Evidence level: cochrane and systematic reviews

aPDT adjunct to SRP with MB or TBO, achieved statistically significant improvements in probing depth (PD) reduction (MD 0.37 mm) and clinical attachment level (CAL) gain (MD 0.33 mm) across a large meta-analysis of 26 randomized controlled trials (RCTs) (34); however, both values fall below the ∼1 mm and ∼0.5 mm thresholds considered clinically meaningful (35). The American Academy of Periodontology (AAP) best evidence review (36) found aPDT comparable to conventional therapy in PD and CAL for periodontitis and peri-implantitis. The European Federation of Periodontology (EFP) clinical practice guideline explicitly recommended not to use aPDT for treatment of stages I-III periodontitis based on low quality of evidence and clinically unmeaningful outcomes (37). The 2024 Cochrane review by Jervøe-Storm et al. supported the findings, indicating negligible clinical significance and identified lack of eligible RCT for peri-implantitis (38). The gap between bactericidal efficacy *in-vitro* and sub-threshold clinical gain could be explained by oxygen dependence of Type II reactions in anaerobic pockets and limited PS diffusion into mature biofilm (13). For peri-implantitis, a 2025 meta-analysis of 26 RCTs reported adjunctive aPDT reductions in probing pocket depth of 0.8–0.9 mm at 3–6 months, with moderate-to-low certainty and high heterogeneity (39); the EFP 2023 S3 guideline nevertheless suggests not to use aPDT in non-surgical peri-implantitis therapy, nor for implant surface decontamination during surgery (32).

### 4.2 Evidence level: in-vitro studies and one clinical trial

To date, no systematic reviews or meta-analyses have evaluated true aBL for periodontal or peri-implant disease. Significant bactericidal effects against key periodontopathogens have been demonstrated *in-vitro*, with 405–460 nm identified as the optimal bactericidal range with the endogenous photosensitizers confirmed as porphyrins and flavins (40–42). Proof-of-concept 405 nm mouthguard and wireless blue-light zirconia implant devices achieved substantial bacterial reduction (43, 44). A clinical trial by Soukos et al., reported significant reduction in *P. gingivalis* (−25%) and *P. intermedia* (−56%) in plaque after 455 nm exposure twice daily for 4 days (45).

### 4.3 Evidence level: overview of systematic reviews

Modeled kill depth for aSP is 2–3 mm, versus 90–150 μm light penetration for aBL and ∼23 μm *in-vitro* biofilm kill for aPDT (8). Chambrone et al. 2018 AAP best evidence review reported Nd:YAG and diode laser adjunctive to SRP produced greater reduction in red complex pathogens at 3–6 months, however, bacterial count-to-clinical outcome gap indicated an insignificant improvement in PD and CAL (46). Subsequent systematic reviews and a 2026 overview of ten reviews confirmed the absence of a clinically relevant adjunctive benefit (47–49). The EFP S3 guideline found insufficient evidence to recommend adjunctive laser therapy of any wavelength (37). For peri-implantitis, the EFP 2023 guideline likewise suggests not to use lasers, adjunctively or as monotherapy (32). Nd:YAG-specific meta-analyses, the closest for the pulsed regime, show similar pattern of modest PD reduction without CAL gain (50, 51), so the sub-threshold result is not explained by CW settings alone.

### 4.4 Evidence level: preclinical studies

PTT for periodontitis and peri-implant disease is almost entirely preclinical, with no clinical trials, or systematic reviews. In periodontitis, AuAg@PC-Fe nanocomposites demonstrated bactericidal activity against periodontal pathogens *in-vitro* following 808 nm activation as well as macrophage activation in a murine periodontitis model (9). In a different study, a thermosensitive injectable hydrogel incorporating T8IC nanoparticles, combined mild PTT (45 °C) with PDT to achieve bactericidal effects alongside osteogenesis induction in a rat periodontitis model (52). No peri-implant-specific PTT study was identified.

## 5 Oral mucosal infections

*Candida albicans* forms a part of the natural oral microflora in healthy adults with a prevalence of 30%–50%. When imbalance occurs, *Candida* can overgrow into a symptomatic form called Oral Candidiasis (53).

### 5.1 Evidence level: systematic reviews

aPDT for oral candidiasis was supported by at least three meta-analyses. Hu et al. (54) reviewed 11 RCTs and reported superior results of aPDT to nystatin in reducing palatal candida (MD −0.87) and comparable to fluconazole (RR 1.01). Al-Maweri et al. (11 RCTs, 460 denture stomatitis patients) and Vila-Nova et al. reported similar findings (55, 56). Across the analyses aPDT consistently achieved outcomes equivalent to conventional antifungals, without inducing resistance—a clinically meaningful advantage. Natural photosensitizers such as riboflavin (vitamin B2), hypericin and curcumin have also shown antifungal activity as low-cost PSs in systematic reviews of mainly *in-vitro* work (57, 58)

### 5.2 Evidence level: in-vitro studies

aBL at 405–450 nm demonstrated significant antifungal activity against *Candida*.

Zhang et al. demonstrated 5.42-log₁₀ killing of *C. albicans* at 415 nm with 70.2 J/cm^2^ *in-vitro*, with only 0.11-log₁₀ viability loss in keratinocytes at the equivalent dose, an approximately 42-fold selectivity (59). 405 nm LED irradiation disinfected *C. albicans*/*S. mutans* dual-species biofilm on denture resin (60). No systematic review or RCT of aBL for oral candidiasis was found.

Beyond candidiasis, aPDT has been examined for herpes labialis in a systematic review that identified case reports only (61) and in two small RCTs where it was found beneficial as an adjunct (62) and matching acyclovir for healing time (63). However, both trials are too small to establish equivalence. No study addresses light-based treatment of bacterial mucosal infections such as angular cheilitis or pericoronitis. aBL inactivates enveloped viruses *in-vitro* (14–16), but no intraoral study was found. Neither aSP nor PTT has been investigated in any oral mucosal infection.

*Candida* lacks the endogenous NIR chromophore required for selective photothermolysis, and PTT-based approaches remain confined to non-oral wound infection models.

## 6 Dental caries

Current recommendation on deep caries removal supports the removal of the infected tissue, and the retention of caries-affected tissue, capable of remineralizing. The recommendation aims to optimize the preservation of the dentin-pulp complex while achieving adequate bond strength for an adhesive restoration (64).

### 6.1 Evidence level: systematic reviews

Focusing on clinical systematic reviews in aPDT, Cieplik et al. (65) identified 6 studies and found significant bacterial load reduction in 5 of them, though heterogeneity precluded meta-analysis. Baghani et al. (66) identified 7 clinical studies and reported significant reductions in total bacterial load, *S. mutans*, and *Lactobacillus* spp. in permanent teeth (SMD 0.64–1.10), while in primary teeth only *S. mutans* reduction reached statistical significance. Similar inconclusive results were found by Kattan on primary teeth (67), reporting bacterial reduction in only half of 8 studies. Regarding clinical outcomes of bond strength, systematic reviews and meta-analyses by Faria et al. (68) and AlFawaz (64) found no significance in adhesive restoration performance and reported that conventional chemical disinfectant provided superior results. Restricted PS diffusion into affected dentin and increased dentinal scattering of light may account for the inconsistent bacterial reduction across studies and the absence of a bond-strength advantage. Lack of clinical translation and inferior results compared to conventional antimicrobials preclude recommendation at this stage.

### 6.2 Evidence level: in-vitro studies

No systematic reviews or clinical trials were found for aBL in dental caries, and all available evidence was extracted from *in-vitro* studies. Mohamad et al. demonstrated *S. mutans* viability reduction on non-carious molars following an exposure to aBL at 405–450 nm mediated through excitation of endogenous flavin chromophores (69). On carious dentin, evidence is limited to a single study by Gomez et al., reporting non-significant results on *S. mutans* biofilm reduction (70).

### 6.3 Evidence level: preclinical studies

No systematic reviews or clinical trials were found for PTT in dental caries. A 2025 scoping review by Xu et al. on nanomaterial-based phototherapy for caries prevention identified only 9 publications, comprising 3 animal studies and 6 laboratory studies (71). *In-vitro* comparison of PTT using indocyanine green against aPDT demonstrated significant CFU reductions in both modalities (72). More advanced nanocomposites including Ag⁺-doped Prussian blue hydrogels, have achieved >99% antibacterial efficacy, with remineralization-promoting effects demonstrated in rat models (73). Clinical recommendations cannot yet be formulated for aBL or PTT.

## 7 Discussion

Two findings characterize the current state of evidence for antimicrobial light-based therapy in dentistry. The first is uneven distribution of research: aPDT is the only intervention with systematic reviews and meta-analyses, whereas aBL and PTT remain *in-vitro* and preclinical despite their mechanistic promise. The second is a gap between microbial and clinical endpoints, which persisted across the interventions: significant reduction in microbial load, but without consistent measurable clinical improvement. aPDT illustrates both (Table 1). The one clear clinical indication for aPDT is oral candidiasis, where it matches conventional antifungals (54–56).

aSP, despite favorable porphyrin selectivity (8) and deeper tissue penetration than aBL or aPDT (8), was no better than conventional irrigation (31) or SRP (46–49), including in Nd:YAG-only analyses (50, 51). Several limitations of light-based interventions are intrinsic to the physical nature of light and tissue structure rather than to the evidence base. These may partially explain the microbial-clinical gap. *E. faecalis*, for example, invades dentinal tubules to several hundred micrometers (19), far surpassing light penetration in aPDT and aBL, and the extracellular matrix further attenuates both light and PS diffusion (8, 13). Other limitations are location specific: the bactericidal properties of aPDT and aBL are oxygen dependent, rendering them less efficient in anaerobic niches of necrotic pulp and deep pockets. In deeper reach interventions such as aSP and PTT other limitations should be taken into consideration such as PDL and pulpal damage resulting from the generated heat. For aSP, Nd:YAG effects on dentinal microcracks should also be considered (31), and for PTT nanoparticle compatibility, clearance and regulatory status remain unresolved. The current state of light-based antimicrobial interventions for dentistry could be described as mechanism-rich, but currently outcome-poor field, without guideline level recommendations. Promising directions for future research are mainly related to aBL and PTT which lack controlled trials. The latter two are supported by mechanism rather than outcome and represent the most promising directions for future research: aBL through its selectivity for porphyrin-rich periodontopathogens (8, 17, 18) and its link to autofluorescence-based diagnostics (18), and PTT through its capacity to combine antimicrobial action with tissue regeneration (9, 52). As a narrative mini-review, this work carries the limitations of single-team selection without a formal systematic protocol or risk-of-bias assessment. Clinical recommendation for any modality will require standardized protocols and adequately powered trials testing whether microbial reduction yields durable clinical benefit.

## References

[1] Naghavi M Vollset SE Ikuta KS Swetschinski LR Gray AP Wool EE. Global burden of bacterial antimicrobial resistance 1990–2021: a systematic analysis with forecasts to 2050. Lancet. (2024) 404(10459):1199–226. 10.1016/S0140-6736(24)01867-1 39299261 PMC11718157

[2] Roberts RM Hersh AL Shapiro DJ Fleming-Dutra KE Hicks LA. Antibiotic prescriptions associated with dental-related emergency department visits. Ann Emerg Med. (2019) 74(1):45–9. 10.1016/j.annemergmed.2018.09.019 30392733 PMC6943909

[3] Peters OA Schönenberger K Laib A. Effects of four Ni-ti preparation techniques on root canal geometry assessed by micro computed tomography. Int Endod J. (2001) 34(3):221–30. 10.1046/j.1365-2591.2001.00373.x 12193268

[4] Sherman PR Hutchens LH Jr Jewson LG Moriarty JM Greco GW McFall WT Jr. The effectiveness of subgingival scaling and root planning. I. Clinical detection of residual calculus. J Periodontol. (1990) 61(1):3–8. 10.1902/jop.1990.61.1.3 2179512

[5] Ferreira PAA Roso LDC Freitas DA Bressani APP Ferreira PHC Bodevan EC. Species identification, virulence factors, and antifungal resistance in clinical *Candida* isolates from ICU patients. Microorganisms. (2026) 14(1):241. 10.3390/microorganisms14010241 41597760 PMC12843725

[6] Hamblin MR Hasan T. Photodynamic therapy: a new antimicrobial approach to infectious disease? Photochem Photobiol Sci. (2004) 3(5):436–50. 10.1039/b311900a 15122361 PMC3071049

[7] Dai T Gupta A Murray CK Vrahas MS Tegos GP Hamblin MR. Blue light for infectious diseases: propionibacterium acnes, helicobacter pylori, and beyond? Drug Resist Updat. (2012) 15(4):223–36. 10.1016/j.drup.2012.07.001 22846406 PMC3438385

[8] Harris DM Sulewski JG. Photoinactivation and photoablation of *Porphyromonas gingivalis*. Pathogens. (2023) 12(9):1160. 10.3390/pathogens12091160 37764967 PMC10535405

[9] Wang P Liang J Liu F. Beyond antibiotics: advances in photothermal strategies for oral infections. Front Bioeng Biotechnol. (2025) 13:1637941. 10.3389/fbioe.2025.1637941 41311979 PMC12647065

[10] Mylona V Anagnostaki E Parker S Cronshaw M Lynch E Grootveld M. Laser-assisted aPDT protocols in randomized controlled clinical trials in dentistry: a systematic review. Dent J (Basel). (2020) 8(3):107. 10.3390/dj8030107 32971996 PMC7558404

[11] Tkaczyk M Mertas A Kuśka-Kiełbratowska A Fiegler-Rudol J Bobela Eż Cisowska M. Assessment of the impact of antimicrobial photodynamic therapy using a 635 nm diode laser and toluidine blue on the susceptibility of selected strains of *Candida* and staphylococcus aureus: an *in vitro* study. Microorganisms. (2025) 13(9):2126. 10.3390/microorganisms13092126 41011459 PMC12472698

[12] Benov L. Photodynamic therapy: current status and future directions. Med Princ Pract. (2015) 24(Suppl 1):14–28. 10.1159/000362416 24820409 PMC6489067

[13] Cieplik F Deng D Crielaard W Buchalla W Hellwig E Al-Ahmad A. Antimicrobial photodynamic therapy—what we know and what we don't. Crit Rev Microbiol. (2018) 44(5):571–89. 10.1080/1040841X.2018.1467876 29749263

[14] Leanse LG Dos Anjos C Mushtaq S Dai T. Antimicrobial blue light: a ‘magic Bullet' For the 21st century and beyond? Adv Drug Deliv Rev. (2022) 180:114057. 10.1016/j.addr.2021.114057 34800566 PMC8728809

[15] Ozdemir GD Dos Anjos C Ozdemir MA Leanse LG Dai T. Lights out for superbugs: is antimicrobial blue light a potential approach for future infection control? Adv Drug Deliv Rev. (2025) 224:115654. 10.1016/j.addr.2025.115654 40651507 PMC12338029

[16] Wang Y Wang Y Wang Y Murray CK Hamblin MR Hooper DC. Antimicrobial blue light inactivation of pathogenic microbes: state of the art. Drug Resist Updat. (2017) 33-35:1–22. 10.1016/j.drup.2017.10.002 29145971 PMC5699711

[17] Yoshida A Sasaki H Toyama T Araki M Fujioka J Tsukiyama K. Antimicrobial effect of blue light using *Porphyromonas gingivalis* pigment. Sci Rep. (2017) 7(1):5225. 10.1038/s41598-017-05706-1 28701797 PMC5507902

[18] Lee ES De Josselin De Jong E Kim BI. Detection of dental plaque and its potential pathogenicity using quantitative light-induced fluorescence. J Biophotonics. (2019) 12(7):e201800414. 10.1002/jbio.201800414 30834691

[19] Stuart CH Schwartz SA Beeson TJ Owatz CB. *Enterococcus faecalis*: its role in root canal treatment failure and current concepts in retreatment. J Endod. (2006) 32(2):93–8. 10.1016/j.joen.2005.10.049 16427453

[20] Lee MT Bird PS Walsh LJ. Photo-activated disinfection of the root canal: a new role for lasers in endodontics. Aust Endod J. (2004) 30(3):93–8. 10.1111/j.1747-4477.2004.tb00417.x 15633797

[21] Chrepa V Kotsakis GA Pagonis TC Hargreaves KM. The effect of photodynamic therapy in root canal disinfection: a systematic review. J Endod. (2014) 40(7):891–8. 10.1016/j.joen.2014.03.005 24935531

[22] Pourhajibagher M Bahador A. Adjunctive antimicrobial photodynamic therapy to conventional chemo-mechanical debridement of infected root canal systems: a systematic review and meta-analysis. Photodiagnosis Photodyn Ther. (2019) 26:19–26. 10.1016/j.pdpdt.2019.02.009 30753922

[23] Quintana RM Scarparo RK Münchow EA Pinheiro LS Tavares CO Kopper PMP. Does aPDT reduce bacterial load in endodontic infected teeth? A systematic review and meta-analysis. Lasers Med Sci. (2023) 38(1):268. 10.1007/s10103-023-03938-4 37981598

[24] Das U Giri J Ayer A. Effectiveness of antimicrobial photodynamic therapy in endodontic infection: a systematic review and meta-analysis. BMC Oral Health. (2026) 26(1):116. 10.1186/s12903-025-07501-1 PMC12817463 41388259

[25] Araújo LP Gobbo LB Silva TA Rosa WLO Almeida JFA Gomes BPFA. Photodynamic therapy in the root canal treatment of primary teeth: a systematic review of clinical trials. Int J Paediatr Dent. (2024) 34(2):114–24. 10.1111/ipd.13088 37195228

[26] American Association of Endodontists. AAE Position Statement: Use of Lasers in Dentistry. Chicago, IL: AAE (2017). Available online at: https://www.aae.org/specialty/wp-content/uploads/sites/2/2017/06/lasersnew.pdf (Accessed September 13, 2026)

[27] Duncan HF El-Karim I. Endodontic S3-level clinical practice guidelines: the European society of endodontology process and recommendations. Br Dent J. (2025) 238(7):580–6. 10.1038/s41415-025-8335-x 40217051 PMC11991915

[28] Zhang L Li Y Zhang Q Du N Li X Zhang Q. Antimicrobial activity of an implantable wireless blue light-emitting diode against root canal biofilm *in vitro*. Photobiomodul Photomed Laser Surg. (2020) 38(11):694–702. 10.1089/photob.2020.4821 33103954

[29] Gutknecht N Al Hassan N Martins MR Conrads G Franzen R. Bactericidal effect of 445-nm blue diode laser in the root canal dentin on *Enterococcus faecalis* of human teeth. Laser Dent Sci. (2018) 2(4):247–54. 10.1007/s41547-018-0044-1

[30] Hendi SS Ahmadyani E Alikhani MY Farhadian M Mirzaei A. Antibacterial effects of the novel blue laser and erbium chromium laser on enterococcus faecalis in root canal dentin with different thicknesses. Int J Dent. (2022) 2022:6119464. 10.1155/2022/6119464 35368314 PMC8975713

[31] Duarte MA Neves JA Caçador M Flores-Fraile J. Clinical efficiency of lasers in endodontic treatment of primary endodontic cases: an umbrella review of systematic reviews. Int Dent J. (2026) 76(3):109470. 10.1016/j.identj.2026.109470 41833582 PMC13000699

[32] Herrera D Berglundh T Schwarz F Chapple I Jepsen S Sculean A. Prevention and treatment of peri-implant diseases—the EFP S3 level clinical practice guideline. J Clin Periodontol. (2023) 50(Suppl 26):4–76. 10.1111/jcpe.13823 37271498

[33] Harrel SK Cobb CM Sheldon LN Rethman MP Sottosanti JS. Calculus as a risk factor for periodontal disease: narrative review on treatment indications when the response to scaling and root planing is inadequate. Dent J (Basel). (2022) 10(10):195. 10.3390/dj10100195 36286005 PMC9600378

[34] Azaripour A Dittrich S Van Noorden CJF Willershausen B. Efficacy of photodynamic therapy as adjunct treatment of chronic periodontitis: a systematic review and meta-analysis. Lasers Med Sci. (2018) 33(2):407–23. 10.1007/s10103-017-2383-7 29177555

[35] Greenstein G. Clinical versus statistical significance as they relate to the efficacy of periodontal therapy. J Am Dent Assoc. (2003) 134(5):583–91. 10.14219/jada.archive.2003.0225 12785493

[36] Chambrone L Wang HL Romanos GE. Antimicrobial photodynamic therapy for the treatment of periodontitis and peri-implantitis: an American academy of periodontology best evidence review. J Periodontol. (2018) 89(7):783–803. 10.1902/jop.2017.170172 30133749

[37] Sanz M Herrera D Kebschull M Chapple I Jepsen S Berglundh T. Treatment of stage I-III periodontitis—the EFP S3 level clinical practice guideline. J Clin Periodontol. (2020) 47(Suppl 22):4–60. 10.1111/jcpe.13290 32383274 PMC7891343

[38] Jervøe-Storm P-M Bunke J Worthington HV Needleman I Cosgarea R MacDonald L. Adjunctive antimicrobial photodynamic therapy for treating periodontal and peri-implant diseases. Cochrane Database Syst Rev. (2024) 2024(7):CD011778. 10.1002/14651858.CD011778.pub2 PMC11240860 38994711

[39] Yan Y Rotundo R Suvan J Orlandi M Poma A D'Aiuto F. Photodynamic therapy and peri-implant diseases: a systematic review and meta-analysis. Front Oral Health. (2025) 6:1614982. 10.3389/froh.2025.1614982 40703980 PMC12283991

[40] Fukui M Yoshioka M Satomura K Nakanishi H Nagayama M. Specific-wavelength visible light irradiation inhibits bacterial growth of *Porphyromonas gingivalis*. J Periodontal Res. (2008) 43(2):174–8. 10.1111/j.1600-0765.2007.01009.x 18302619

[41] Cieplik F Späth A Leibl C Gollmer A Regensburger J Tabenski L. Blue light kills *Aggregatibacter actinomycetemcomitans* due to its endogenous photosensitizers. Clin Oral Investig. (2014) 18(7):1763–9. 10.1007/s00784-013-1151-8 24297656

[42] Fontana CR Song X Polymeri A Goodson JM Wang X Soukos NS. The effect of blue light on periodontal biofilm growth *in vitro*. Lasers Med Sci. (2015) 30(8):2077–86. 10.1007/s10103-015-1724-7 25759232

[43] Souza MA Corralo DJ Gabrielli ES Figueiredo JAP Cohen S Wolff M. Oral bacterial decontamination using an innovative prototype for photocatalytic disinfection. Clin Oral Investig. (2022) 26(3):3005–10. 10.1007/s00784-021-04282-z 34775518

[44] Zhang L Li Y Yuan L Zhang Q Yan Y Dong F. Advanced and readily-available wireless-powered blue-light-implant for non-invasive peri-implant disinfection. Adv Sci (Weinh). (2023) 10(14):e2203472. 10.1002/advs.202203472 36935373 PMC10190665

[45] Soukos NS Stultz J Abernethy AD Goodson JM. Phototargeting human periodontal pathogens *in vivo*. Lasers Med Sci. (2015) 30(3):943–52. 10.1007/s10103-013-1497-9 24346334

[46] Chambrone L Ramos UD Reynolds MA. Infrared lasers for the treatment of moderate to severe periodontitis: an American academy of periodontology best evidence review. J Periodontol. (2018) 89(7):743–65. 10.1902/jop.2017.160504 29682757

[47] Patel S Awan KH Freitas CMT Bhandi S Licari FW Patil S. Diode laser targeting red-complex bacteria in periodontitis: a systematic review. Eur Rev Med Pharmacol Sci. (2023) 27(24):11806–16. 10.26355/eurrev_202312_34779 38164844

[48] Zhao P Song X Nie L Wang Q Zhang P Ding Y. Efficacy of adjunctive photodynamic therapy and lasers in the non-surgical periodontal treatment: a Bayesian network meta-analysis. Photodiagnosis Photodyn Ther. (2020) 32:101969. 10.1016/j.pdpdt.2020.101969 32846208

[49] Typou P Neophytou C Papadimitriou K. The role of lasers in non-surgical periodontal treatment. Cureus. (2026) 18(2):e102961. 10.7759/cureus.102961 41798431 PMC12965296

[50] Sgolastra F Severino M Petrucci A Gatto R Monaco A. Nd:yAG laser as an adjunctive treatment to nonsurgical periodontal therapy: a meta-analysis. Lasers Med Sci. (2014) 29(3):887–95. 10.1007/s10103-013-1293-6 23474742

[51] Atya A Tawfik A Waly R Abdelazim K El Aziz Elsandoby ASIA Wadan A-HS. Efficacy of adjunct nd-YAG laser (1064 nm) to non-surgical periodontal therapy: a systematic review and meta-analysis of randomized controlled trials. Photochem Photobiol Sci. (2026) 25:1997–2015. 10.1007/s43630-026-00962-5 42560618

[52] Wang W Zhang G Wang Y Ran J Chen L Wei Z. An injectable and thermosensitive hydrogel with nano-aided NIR-II phototherapeutic and chemical effects for periodontal antibacteria and bone regeneration. J Nanobiotechnology. (2023) 21(1):367. 10.1186/s12951-023-02124-6 37805588 PMC10559606

[53] Lu SY. Oral candidosis: pathophysiology and best practice for diagnosis, classification, and successful management. J Fungi (Basel). (2021) 7(7):555. 10.3390/jof7070555 34356934 PMC8306613

[54] Hu Q Li T Yang J Peng Y Liu Q Liu N. Efficacy of photodynamic therapy in the treatment of oral candidiasis: a systematic review and meta-analysis. BMC Oral Health. (2023) 23(1):802. 10.1186/s12903-023-03484-z 37884914 PMC10605952

[55] Al-Maweri SA Alhajj MN Anweigi L Ashraf S Halboub E Salleh NM. Efficacy of photodynamic therapy on candida colonization and clinical symptoms in denture stomatitis: a systematic review and meta-analysis. BMC Oral Health. (2024) 24(1):84. 10.1186/s12903-023-03789-z 38229054 PMC10790471

[56] Vila-Nova TEL Leão RS Santiago Junior JF Pellizzer EP Vasconcelos BCDE Moraes SLD. Photodynamic therapy in the treatment of denture stomatitis: a systematic review and meta-analysis. J Prosthet Dent. (2023) 130(6):825–32. 10.1016/j.prosdent.2021.11.028 35125209

[57] Łopaciński M Fiegler-Rudol J Niemczyk W Skaba D Wiench R. Riboflavin- and hypericin-mediated antimicrobial photodynamic therapy as alternative treatments for oral candidiasis: a systematic review. Pharmaceutics. (2025) 17(1):33. 10.3390/pharmaceutics17010033 PMC11768293 39861682

[58] Kubizna M Dawiec G Wiench R. Efficacy of curcumin-mediated antimicrobial photodynamic therapy on *Candida* spp.—a systematic review. Int J Mol Sci. (2024) 25(15):8136. 10.3390/ijms25158136 39125706 PMC11311843

[59] Zhang Y Zhu Y Chen J Wang Y Sherwood ME Murray CK. Antimicrobial blue light inactivation of *Candida albicans*: *in vitro* and *in vivo* studies. Virulence. (2016) 7(5):536–45. 10.1080/21505594.2016.1155015 26909654 PMC5026794

[60] Tsutsumi-Arai C Arai Y Terada-Ito C Imamura T Tatehara S Ide S. Microbicidal effect of 405-nm blue LED light on *Candida albicans* and *Streptococcus mutans* dual-species biofilms on denture base resin. Lasers Med Sci. (2022) 37(2):857–66. 10.1007/s10103-021-03323-z 33931832

[61] Lotufo MA Tempestini Horliana ACR Santana T De Queiroz AC Gomes AO Motta LJ. Efficacy of photodynamic therapy on the treatment of herpes labialis: a systematic review. Photodiagnosis Photodyn Ther. (2020) 29:101536. 10.1016/j.pdpdt.2019.08.018 31648056

[62] Ajmal M. Effectiveness of photodynamic therapy as an adjunct to topical antiviral therapy in the treatment of herpes labialis: a randomized controlled clinical trial. Photodiagnosis Photodyn Ther. (2021) 34:102302. 10.1016/j.pdpdt.2021.102302 33894370

[63] La Selva A Bezerra DT Zerbinati RM Gonçalves MLL Navarro RS Bello-Silva MS. Herpes simplex treated with antimicrobial photodynamic therapy: randomized controlled trial. J Oral Pathol Med. (2025) 54(7):547–55. 10.1111/jop.13647 40485092

[64] AlFawaz YF. Adhesive bond strength of restorative materials to caries-affected dentin treated with antimicrobial photodynamic therapy: a systematic review and meta-analysis. Photobiomodul Photomed Laser Surg. (2024) 42(2):125–39. 10.1089/photob.2023.0037 38011333

[65] Cieplik F Buchalla W Hellwig E Al-Ahmad A Hiller KA Maisch T. Antimicrobial photodynamic therapy as an adjunct for treatment of deep carious lesions—a systematic review. Photodiagnosis Photodyn Ther. (2017) 18:54–62. 10.1016/j.pdpdt.2017.01.005 28099873

[66] Baghani Z Karrabi M Assarzadeh H. Dentition type as a determinant of microbial load reduction by antimicrobial photodynamic therapy in deep dentin caries: a systematic review and meta-analysis. J Dent (Shiraz). (2024) 25(4):296–308. 10.30476/dentjods.2023.98616.2098 39713109 PMC11662176

[67] Kattan HF. Efficacy of antimicrobial photodynamic therapy (aPDT) in reducing cariogenic bacteria in primary deciduous dentine. Photodiagnosis Photodyn Ther. (2021) 36:102600. 10.1016/j.pdpdt.2021.102600 34699983

[68] Faria LV Fernandes TO Guimarães LS Cajazeira MRR Antunes LS Antunes LAA. Does selective caries removal in combination with antimicrobial photodynamic therapy affect the clinical performance of adhesive restorations of primary or permanent teeth? A systematic review with meta-analysis. J Clin Pediatr Dent. (2022) 46(5):1–14. 10.22514/jocpd.2022.002 36624909

[69] Mohamad SA Megson IL Kean AH. Blue light photoinhibition of *Streptococcus mutans*: potential chromophores and mechanisms. Laser Dent Sci. (2023) 7(4):195–205. 10.1007/s41547-023-00204-2

[70] Felix Gomez GG Lippert F Ando M Zandona AF Eckert GJ Gregory RL. Photoinhibition of *Streptococcus mutans* biofilm-induced lesions in human dentin by violet-blue light. Dent J (Basel). (2019) 7(4):113. 10.3390/dj7040113 31835833 PMC6960986

[71] Xu VW Yin IX Niu JY Chu CH. Enhancing caries preventive effects of nanomaterials with phototherapy: a scoping review. J Funct Biomater. (2025) 16(9):308. 10.3390/jfb16090308 41003379 PMC12471128

[72] Beytollahi L Pourhajibagher M Chiniforush N Ghorbanzadeh R Raoofian R Pourakbari B. The efficacy of photodynamic and photothermal therapy on biofilm formation of *Streptococcus mutans*: an *in vitro* study. Photodiagnosis Photodyn Ther. (2017) 17:56–60. 10.1016/j.pdpdt.2016.10.006 27769914

[73] Li S Li Q Zhang H Li F Hu J Qian J. Dental caries management with antibacterial silver-doped prussian blue hydrogel by the combined effects of photothermal response and ion discharge. ACS Appl Mater Interfaces. (2024) 16(22):28172–83. 10.1021/acsami.4c04302 38772043

